# Identification of a cholesterol metabolism-related prognostic signature for multiple myeloma

**DOI:** 10.1038/s41598-023-46426-z

**Published:** 2023-11-08

**Authors:** Na Zhao, Chunxia Qu, Yan Yang, Huihui Li, Yueyue Li, Hongbo Zhu, Zhiguo Long

**Affiliations:** 1 Department of Hematology, Shanghai Pudong Hospital, Fudan University, Shanghai, 201399 China; 2 Department of Pathology, Shanghai Pudong Hospital, Fudan University, Shanghai, 201399 China

**Keywords:** Cancer, Computational biology and bioinformatics, Metabolomics

## Abstract

Multiple myeloma (MM) is a prevalent hematological malignancy that poses significant challenges for treatment. Dysregulated cholesterol metabolism has been linked to tumorigenesis, disease progression, and therapy resistance. However, the correlation between cholesterol metabolism-related genes (CMGs) and the prognosis of MM remains unclear. Univariate Cox regression analysis and LASSO Cox regression analysis were applied to construct an overall survival-related signature based on the Gene Expression Omnibus database. The signature was validated using three external datasets. Enrichment analysis and immune analysis were performed between two risk groups. Furthermore, an optimal nomogram was established for clinical application, and its performance was assessed by the calibration curve and C-index. A total of 6 CMGs were selected to establish the prognostic signature, including ANXA2, CHKA, NSDHL, PMVK, SCAP and SQLE. The prognostic signature demonstrated good prognostic performance and correlated with several important clinical parameters, including number of transplants, International Staging System, albumin, beta2-Microglobulin and lactate dehydrogenase levels. The function analysis and immune analysis revealed that the metabolic pathways and immunologic status were associated with risk score. The nomogram incorporating the signature along with other clinical characteristics was constructed and the discrimination was verified by the calibration curve and C-index. Our findings indicated the potential prognostic connotation of cholesterol metabolism in MM. The development and validation of the prognostic signature is expected to aid in predicting prognosis and guiding precision treatment for MM.

## Introduction

Multiple myeloma (MM) is a hematologic malignancy that arises from the heterogeneous clonal proliferation of plasma cells and accounts for over 17% of all cases in this category^1^. While the emergence of novel therapies like immunotherapy, monoclonal antibodies, and proteasome inhibitors has led to a significant improvement in MM survival rates over the past 15 years, the disease remains incurable due to high rates of recurrence and mortality^2–4^. For example, bortezomib, a reversible inhibitor of proteasome, has been applied in MM patients for several years^5^. However, while bortezomib effectively eliminates bortezomib-sensitive tumor subclones, it unfortunately also stimulates the growth and expansion of bortezomib-resistant subclones, which contributes to the relapse of MM^6,7^. Thus, it’s important to develop new biomarkers and effective models to stratify the risk and prognosis of MM, as well as guide the individual treatment.

Metabolic reprogramming has emerged as an important aspect of cancer, and dysregulated cholesterol metabolism has been identified as an integral part of tumor proliferation, invasion, and metastasis^8,9^. Cholesterol is an essential component of mammalian membrane structure and plays a crucial role in maintaining cell homeostasis and critical cellular structure^10–12^. Several genes related to cholesterol metabolism have been found to be dysregulated in tumor samples, with LDLR overexpression, which mediates cholesterol uptake, being associated with the development and occurrence of most cancer cells^13–15^. Additionally, various transcription factors that regulate cholesterol metabolism, including SREBP2 and RORγ, have been found to be upregulated in different tumors such as colon cancer, breast cancer, and glioblastoma^16–18^. The proliferation and survival of MM cells are also found to be correlated with cholesterol metabolism. Liver X receptors (LXR), a transcriptional regulator that regulates lipid and cholesterol homeostasis, could influence clonogenic tumor growth and self-renewal potential in MM^19^. 5,6-epoxycholesterol isomers, a kind of cholesterol, has been found to induce oxiapoptophagy and demonstrate anti-tumor activity against MM cells^20^. Previous researches have also demonstrated that high-dose statins could suppress MM cell survival through inhibition of cholesterol biosynthesis^21,22^. Furthermore, Recent studies have shown that cholesterol can regulate immune cells within the tumor microenvironment, playing a role in T cell exhaustion and macrophage polarization and influencing the efficacy of cancer immunotherapy^23–25^. However, there is currently no relevant research on the prognostic value of cholesterol metabolism-related genes (CMGs) in MM.

The aim of this study is to develop a prognostic signature based on CMGs and explore its significance in MM patients. We validated the predictive value of our signature using both training cohort and three external validation cohorts. Additionally, we investigated the connection between the signature and functional signaling pathways, as well as immune status. Our findings are expected to help the diagnosis and precision treatment of MM.

## Methods

### Study population and data acquisition

Our study included five cohorts of MM patients obtained from the Gene Expression Omnibus (GEO) database (https://www.ncbi.nlm.nih.gov/geo/) with accession numbers GSE136324, GSE136337, GSE118985, GSE24080, and GSE4204. The microarray expression data and detailed clinical information were downloaded. We excluded samples if corresponding survival data were missing. The GSE136324 dataset served as the training cohort, while the GSE136337, GSE24080, and GSE4204 datasets were used as validation cohorts. The GSE118985 dataset was used to compare the expression of CMGs between tumor and normal samples.

### Collection of cholesterol metabolism-associated genes

Five genesets related to cholesterol metabolism, including Hallmark cholesterol homeostasis genes, GOBP regulation of cholesterol genes, WP cholesterol metabolism genes, WP cholesterol biosynthesis genes and Reactome cholesterol biosynthesis genes were obtained from the Molecular Signature Database (https://www.gsea-msigdb.org/gsea/msigdb/index.jsp)^26,27^. A total of 140 CMGs were collected after removing the overlapping genes from the aforementioned five genesets. After intersecting the 140 CMGs with all the genes included in the five MM datasets, we retrieved 123 reliable CMGs for further analysis (Supplementary Fig. 1 and Supplementary Table 1).

### Construction of prognostic signature

Univariate Cox hazards regression analysis was applied to identify CMGs that had a significant correlation with overall survival (OS) in the GSE136324 and GSE136337 datasets (*P* < 0.05). Then, we used the least absolute shrinkage and selection operator (LASSO) regression analysis to determine the crucial signatures and corresponding coefficients for model construction with 1000-fold cross-validation based on the eligible OS-related CMGs^28^. The cholesterol metabolism index (CMI) could be calculated using the following formula:

$$\mathrm{CMI}=\sum \beta i*Ei$$

βi represents the corresponding regression coefficient while Ei represents the expression level of each gene. The CMI was then normalized and transformed by subtracting the minimum value in each dataset and dividing by the maximum value, allowing CMI to map to the range of 0–1. Based on the median cutoff of CMI, patients were divided into high and low-risk groups.

### Assessment of drug responsiveness

The “OncoPredict” package was used to assess the drug susceptibility^29^. The transcriptomic and cell line response data from the Sanger’s Genomics of Drug Sensitivity in Cancer (GDSC) was downloaded and applied as training cohort^30^.

### Differential gene analysis and functional enrichment analysis

The differentially expressed genes (DEGs) were identified using Limma with the significance criteria set to |log2FC|> 0.4 and false discovery rate (FDR) < 0.05. Gene set enrichment analysis (GSEA) was then performed to explore the enriched signaling pathways and intrinsic functions. The GSEA software (version 4.3.2, available at http://software.broadinstitute.org/gsea) with the “h.all.v7.5.1.entrez.gmt” and “msigdb.v7.5.1.entrez.gmt” molecular signature databases was utilized, with FDR < 0.05 considered statistically significant^26^. One thousand total permutations were applied to ensure the validity of the results. Additionally, functional protein–protein interaction network analysis was conducted using STRING^31^.

### Estimation of immune cell infiltration

Cell-type Identification By Estimating Relative Subsets Of RNA Transcripts (CIBERSORT) was used to analyze immune cell infiltration in the high and low-risk groups based on LM22 signatures with 1000 permutations^32^.

### Development and validation of cholesterol metabolism-correlated clinicopathologic nomogram

Univariate and multivariate Cox regression analyses were conducted to determine whether CMI was an independent prognostic predictor of MM. Based on the results of these analyses, a cholesterol metabolism-correlated clinicopathologic nomogram was developed by incorporating CMI with five other clinical characteristics, using the R packages “cmprsk” and “rms”. To assess the predictive discrimination of the nomogram, calibration curves and concordance indices (C-index) were calculated^33,34^. In a well-calibrated model, the predictions of the calibration curve are expected to fall on a 45° diagonal line, and the C-index ranges from 0.5 to 1.0, with 0.5 indicating random chance and 1.0 indicating perfect discrimination.

### Statistical analysis

The statistical analyses were conducted using R software (version 4.2.1, http://www.R-project.org) and relevant packages. The Kaplan–Meier method was employed to generate survival curves, and the log-rank test was used to compare them. Univariate and multivariate Cox regression analyses were conducted to identify OS-related CMGs and independent prognostic indicators of OS. When appropriate, the Wilcoxon test and Kruskal–Wallis test were used to compare continuous variables across different groups. Spearman’s correlation test was applied for correlation analysis. A two-sided *P*-value of less than 0.05 was considered statistically significant. The false discovery rate correction was used for multiple tests to reduce the false-positive rate.

## Results

### Establishment of prognostic signature based on cholesterol metabolism-associated genes

**Figure 1:**
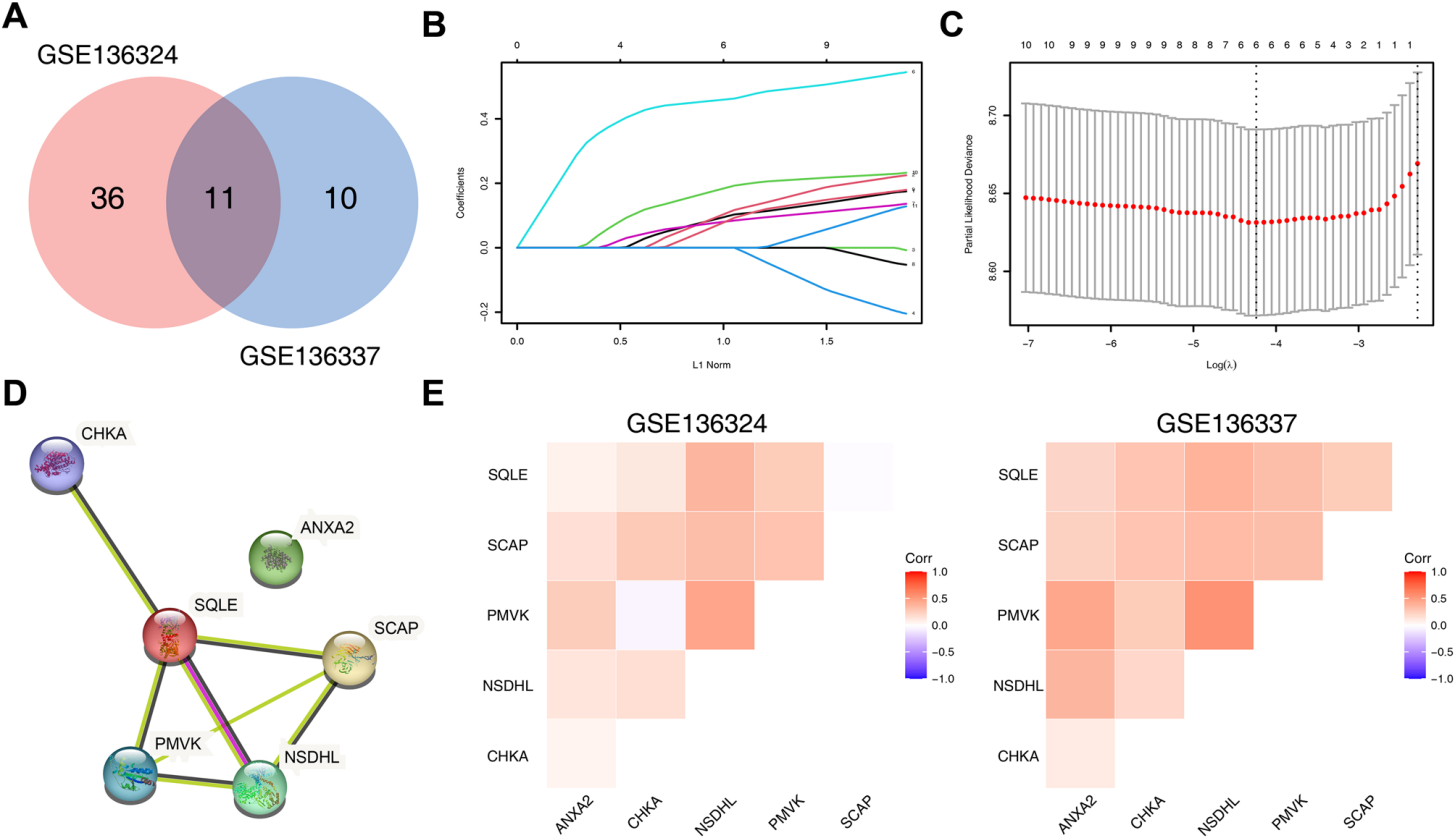
Establishment of prognostic signature based on cholesterol metabolism-associated genes. (**A**) Venn diagram to identify overlapping prognostic CMGs between GSE136324 and GSE136337 (**B**) Coefficient profiles of LASSO Cox regression analysis. (**C**) Partial likelihood deviance for LASSO Cox regression analysis. (**D**) The protein–protein interaction network of CMGs included in the signature. (**E**) The correlation matrix plot displaying the correlation features among CMGs included in the signature.

To determine the potential prognostic value of each available CMG, we utilized the univariate Cox hazards regression analysis in the GSE136324 and GSE136337 datasets to identify prognostic relevance of CMGs, resulting in 47 and 21 significant OS-related CMGs, respectively (Supplementary Table 2). By intersecting the results of the two cohorts, we identified 11 overlapping OS-related CMGs that were eligible for further analysis (Fig. 1A). LASSO Cox regression model was further performed in the GSE136324 cohort aiming to unearth the optimal CMGs for establishing the prognostic signature. Ultimately, we identified six genes to construct the signature, including ANXA2, CHKA, NSDHL, PMVK, SCAP and SQLE (Fig. 1B,C). The CMI of each patient was calculated using the signature formula: CMI = 0.0949 * ANXA2 + 0.1037 * CHKA + 0.4595 * NSDHL + 0.0803 * PMVK + 0.0783 * SCAP. The protein–protein interaction network of these genes revealed that SQLE was the hub gene (Fig. 1D and Supplementary Table 3). Moreover, the expression of most signature genes was significantly correlated with each other (Fig. 1E and Supplementary Fig. 2).

### Verifying the expression and prognostic capability of signature-containing genes

**Figure 2:**
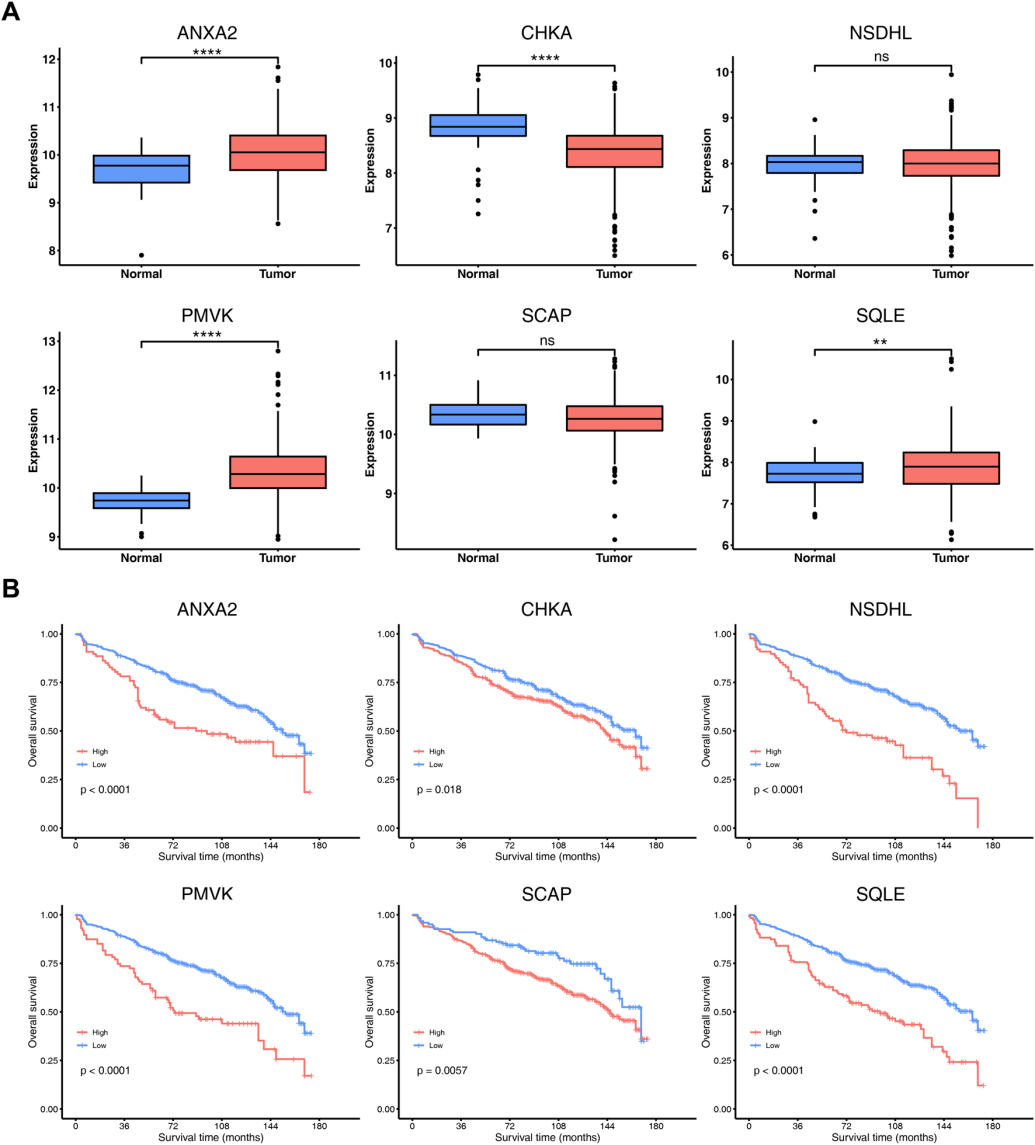
Validation of the expression and prognostic capability of signature-containing genes. (**A**) Boxplot showing the expression difference of 6 CMGs between tumor samples and normal samples in GSE118985. (**B**) Kaplan–Meier analyses of overall survival based on expression levels of 6 CMGs in GSE136324. **, *P* < 0.01; ****, *P* < 0.0001; ns, not significant.

Figure 2A shows the validation of the expression levels of the 6 CMGs in normal and MM tissues using the GSE118985 dataset. The results indicated that ANXA2, PMVK and SQLE were significantly upregulated in MM samples, while CHKA was downregulated. However, NSDHL and SCAP did not show significant changes in expression. Besides, in the separated survival analyses of OS, it was observed that MM patients with high expression of all selected genes had worse prognoses compared to those with low expression in all four datasets, except that SCAP in the GSE4204 did not reach the statistical significance (Fig. 2B and Supplementary Fig. 3).

### Assessment and validation of prognostic signature

**Figure 3:**
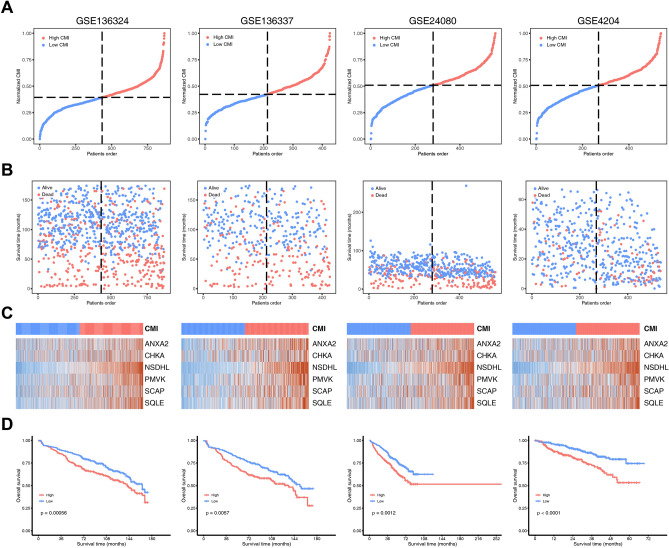
Assessment and validation of prognostic signature in the training and validation cohorts. (**A**) Distribution of the patients’ normalized CMI. (**B**) Scatter plots of patients’ overall survival time and their CMI. (**C**) Heatmaps showing the expression levels of 6 CMGs in MM samples. (**D**) Kaplan–Meier curves showing overall survival of MM patients in the high- and low-CMI groups.

In the GSE136324 dataset, we categorized MM patients into high- and low-CMI groups based on the median threshold of CMI (Fig. 3A). The proportion of deceased patients was higher in the high-CMI group than in the low-CMI group (Fig. 3B). Furthermore, as CMI increased, expression levels of all signature genes also increased (Fig. 3C). Patients with MM in the high-CMI group had significantly worse overall survival compared to those in the low-CMI group (*P* = 0.00056) (Fig. 3D). To confirm these findings' prognostic significance for CMI, we conducted similar analyses using three external validation datasets: GSE136337, GSE24080 and GSE4204. Using an identical calculation formula for CMI categorization into high- and low-groups among MM patients revealed that consistent with our previous results from GSE136324 dataset; patients belonging to a high-CMI group experienced worse OS than those belonging to a low-CMI group across all validation cohorts (Fig. 3D). Furthermore, we found that MM patients in the high-CMI group had worse progression-free survival (PFS) in the GSE136324 and GSE136337 datasets, while there was only trend for significant difference of event-free survival (EFS) in the GSE24080 dataset (Supplementary Fig. 4).

### Integrated analysis of prognostic signature and clinicopathologic factors

**Figure 4:**
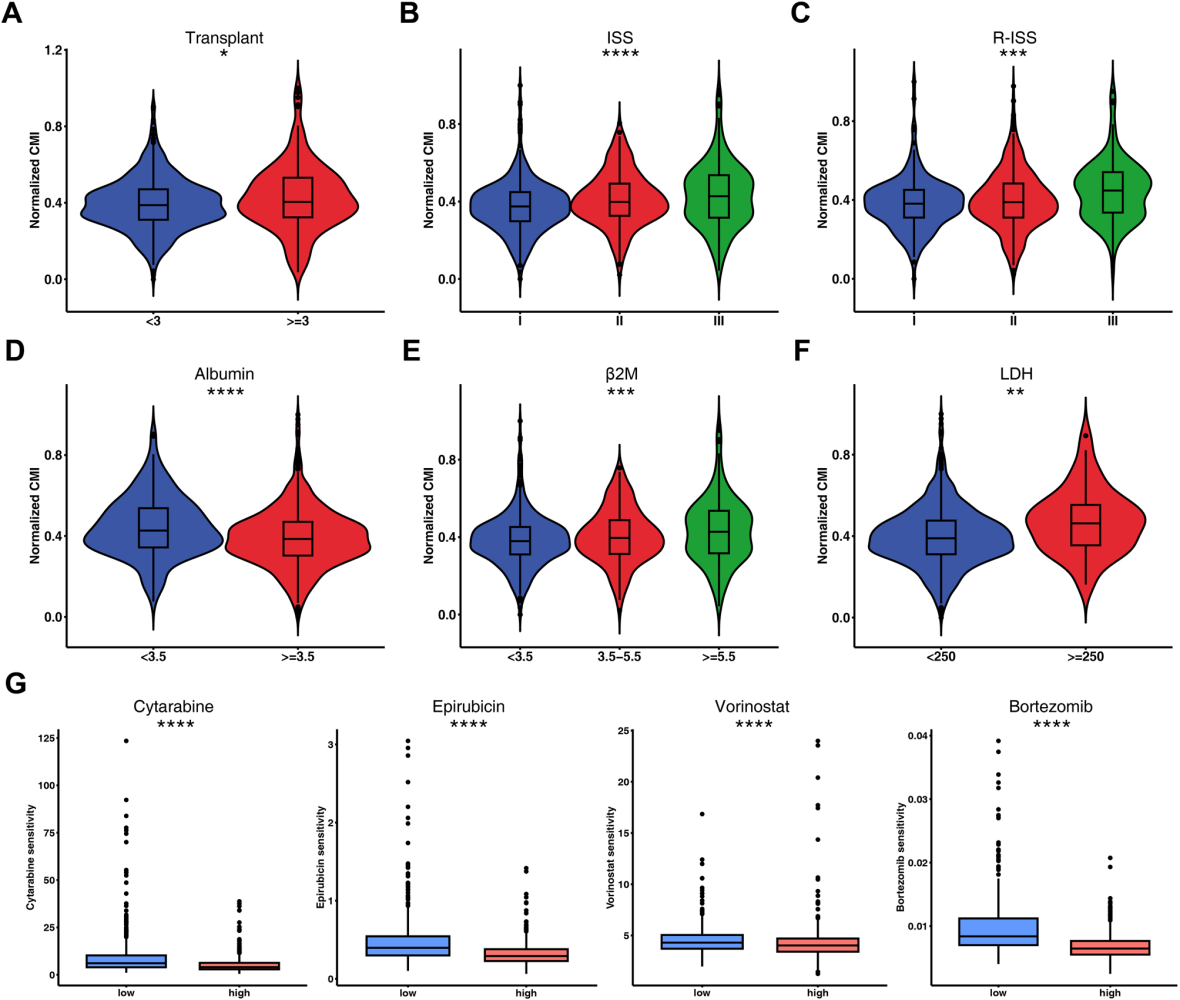
Integrated analysis of prognostic signature and clinicopathologic factors. Comparison of normalized CMI according to (**A**) number of transplants, (**B**) International Staging System (ISS), (**C**) revised-ISS (R-ISS), (**D**) albumin levels, (**E**) beta-2 microglobulin (β2M) levels and (**F**) lactate dehydrogenase (LDH) levels. (**G**) Evaluations of the drug susceptibility between high- and low-CMI groups in the GSE136324 dataset. *, *P* < 0.05; **, *P* < 0.01; ***, *P* < 0.001; ****, *P* < 0.0001.

We examined the relationship between CMI and clinicopathologic characteristics. Significant differences in CMI were observed across various clinical parameters, such as number of transplants, International Staging System (ISS), revised-ISS (R-ISS), albumin levels, beta-2 microglobulin (β2M) levels, and lactate dehydrogenase (LDH) levels (Fig. 4A–F). Our findings suggested that higher CMI was associated with higher β2M and LDH levels, ISS and R-ISS staging, as well as lower albumin levels. However, we did not observe any correlation between CMI and age, gender or race (Supplementary Fig. 5). The result was further confirmed using the GSE24080 dataset (Supplementary Fig. 6). Moreover, we observed that higher CMI was correlated with GEP-70 high risk status, deletion (del)1p32, del1p, del1q, del13q and del16q (Supplementary Fig. 7). We also estimated the drug response to the chemotherapeutic agents and found that the high-CMI cohort showed more sensitive to cytarabine, epirubicin, vorinostat and bortezomib compared to the low-CMI group (Fig. 4G and Supplementary Fig. 8).

### Functional analysis of prognostic signature

**Figure 5:**
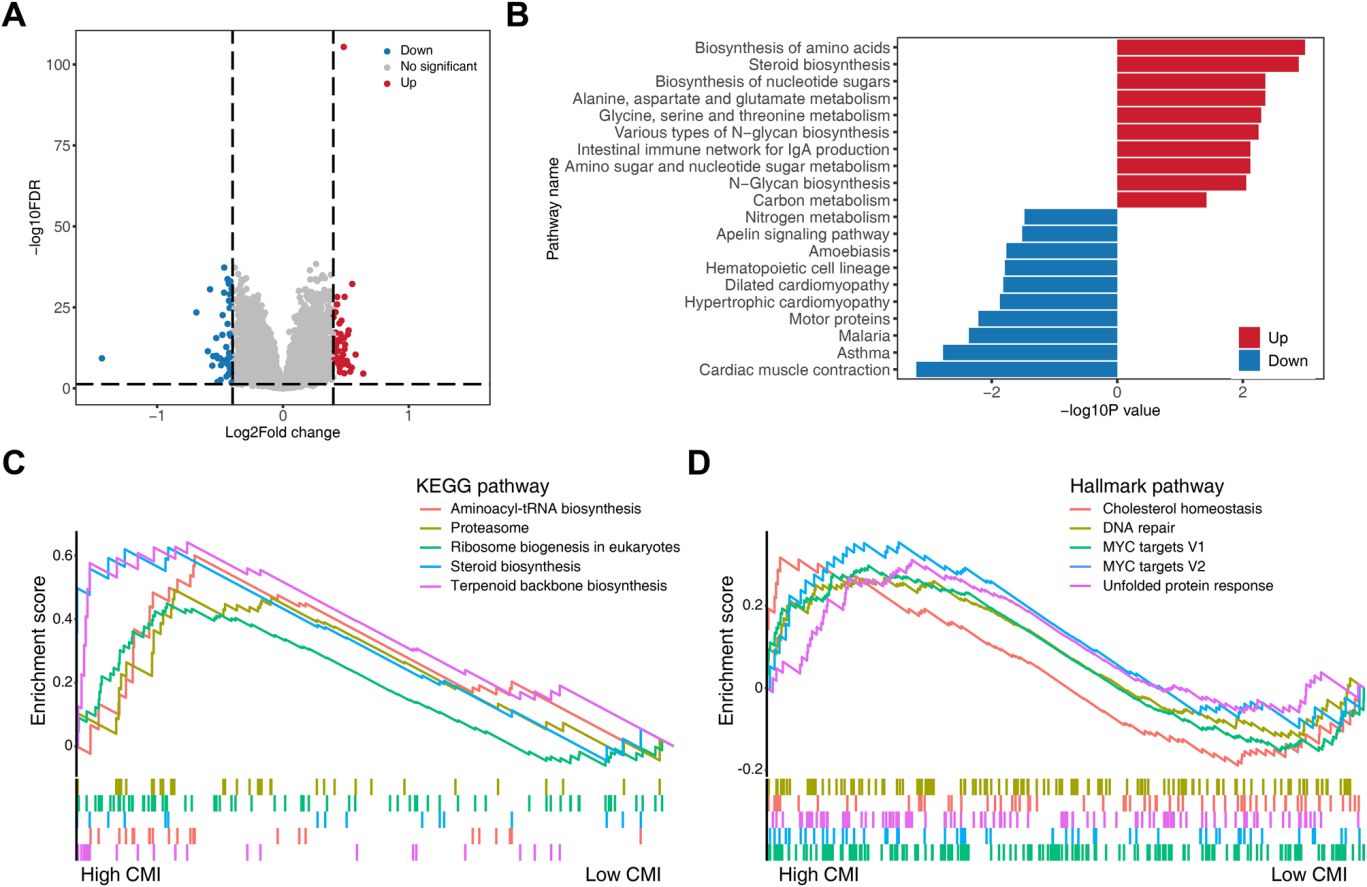
Functional analysis of prognostic signature using GSE136324 dataset. (**A**) Volcano plot showing differentially expressed genes between high- and low-CMI groups. (**B**) Upregulated and downregulated pathways in the high-CMI group based on KEGG pathway enrichment analysis of differentially expressed genes. (**C**, **D**) Summary of GSEA results according to KEGG pathway and hallmark pathway.

We screened for differentially expressed genes between high- and low-CMI groups in the GSE136324 database. We identified a total of 98 DEGs, with 45 significantly upregulated genes and 47 essentially downregulated genes in the high-CMI group (Fig. 5A). Kyoto Encyclopedia of Genes and Genomes (KEGG) enrichment analysis showed that the high-CMI group was mainly enriched in metabolic pathways including biosynthesis of amino acids, steroid biosynthesis and biosynthesis of nucleotide sugars while the low-CMI group was mainly enriched in motor proteins (Fig. 5B)^35^. The results of GSEA analysis using the KEGG database demonstrated that aminoacyle-tRNA biosynthesis, proteasome, ribosome biogenesis in eukaryotes, steroid biosynthesis and terpenoid backbone biosynthesis were significantly enriched in the high-CMI group, while cholesterol homeostasis, DNA repair, MYC targets and unfolded protein response were fundamentally abundant in the high-CMI group through the GSEA results using the hallmark database (Fig. 5C,D and Supplementary Fig. 9).

### Immune associated analysis of prognostic signature

**Figure 6:**
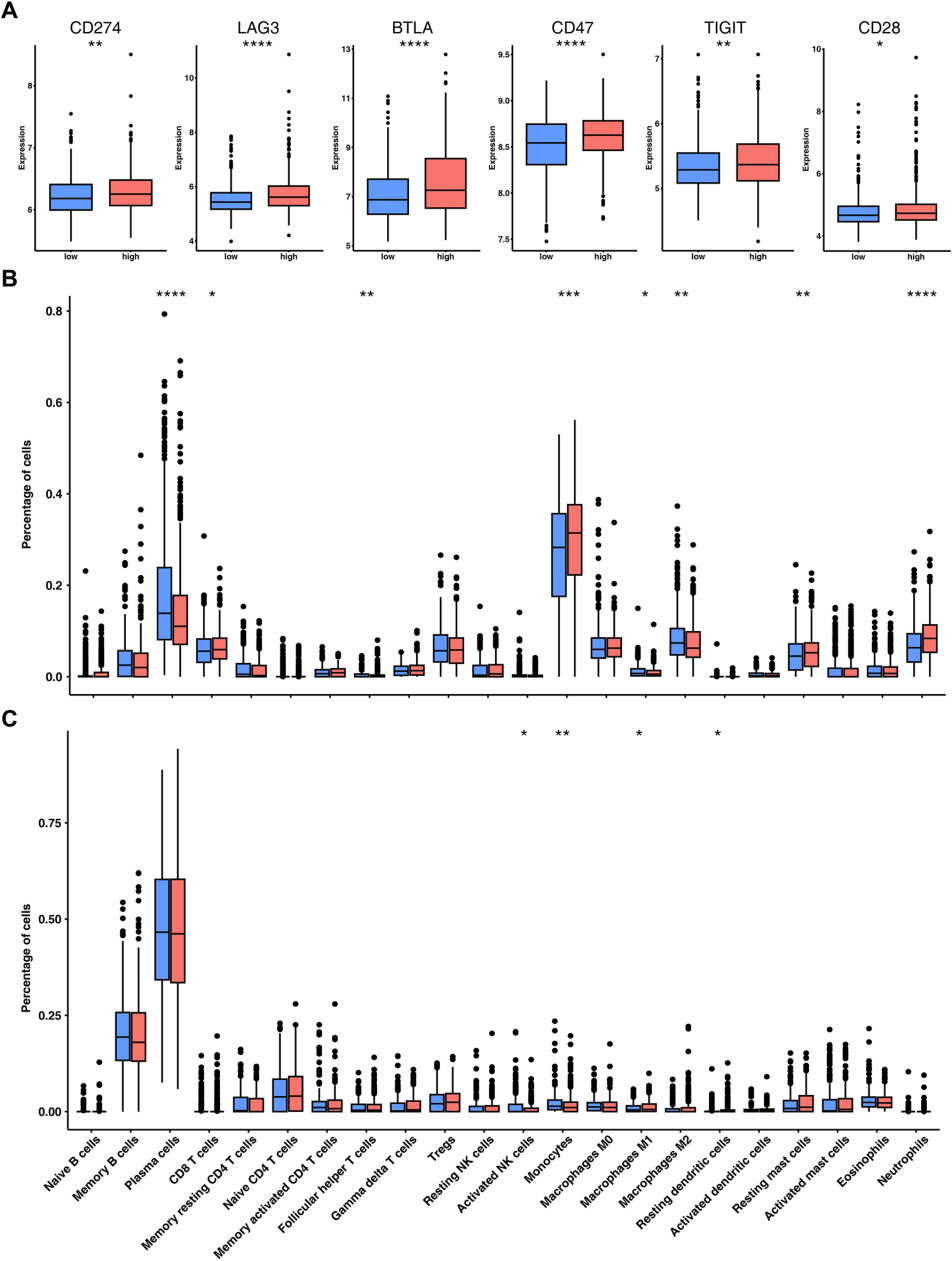
Immune analysis of prognostic signature. (**A**) Boxplot showing the comparison of several immune checkpoints between high- and low-CMI groups. (**B**, **C**) Boxplot showing the comparison of 22 different immune cell types estimated by CIBERSORT between high- and low-CMI groups in (**B**) GSE136324 and (**C**) GSE24080 datasets. *, *P* < 0.05; **, *P* < 0.01; ***, *P* < 0.001; ****, *P* < 0.0001.

Immune checkpoint inhibitors have been developed and evaluated for various types of cancer. In this study, we compared the expression levels of several immune checkpoints between low- and high-CMI groups. Our findings indicate that CD274 (PD-L1), LAG3, BTLA, CD47, TIGIT and CD28 were significantly upregulated in the high-CMI group (Fig. 6A). Additionally, we used the CIBERSORT algorithm to evaluate 22 distinct types of tumor-infiltrating immune cells in low- and high-CMI patients. The results showed that abundance of macrophages M1 subtype were significantly lower in the high-CMI group in all four datasets (Fig. 6B,C and Supplementary Fig. 10). High-CMI group also had significantly less resting dendritic cells in three datasets. Furthermore, there were less CD8^+^ T cells and neutrophils in the High-CMI patients in the GSE136324 and GSE136337 datasets, while more monocytes and activated NK cells were found in the High-CMI patients in the GSE24080 and GSE4204 datasets.

### Development and evaluation of cholesterol metabolism-correlated clinicopathologic nomogram

**Figure 7:**
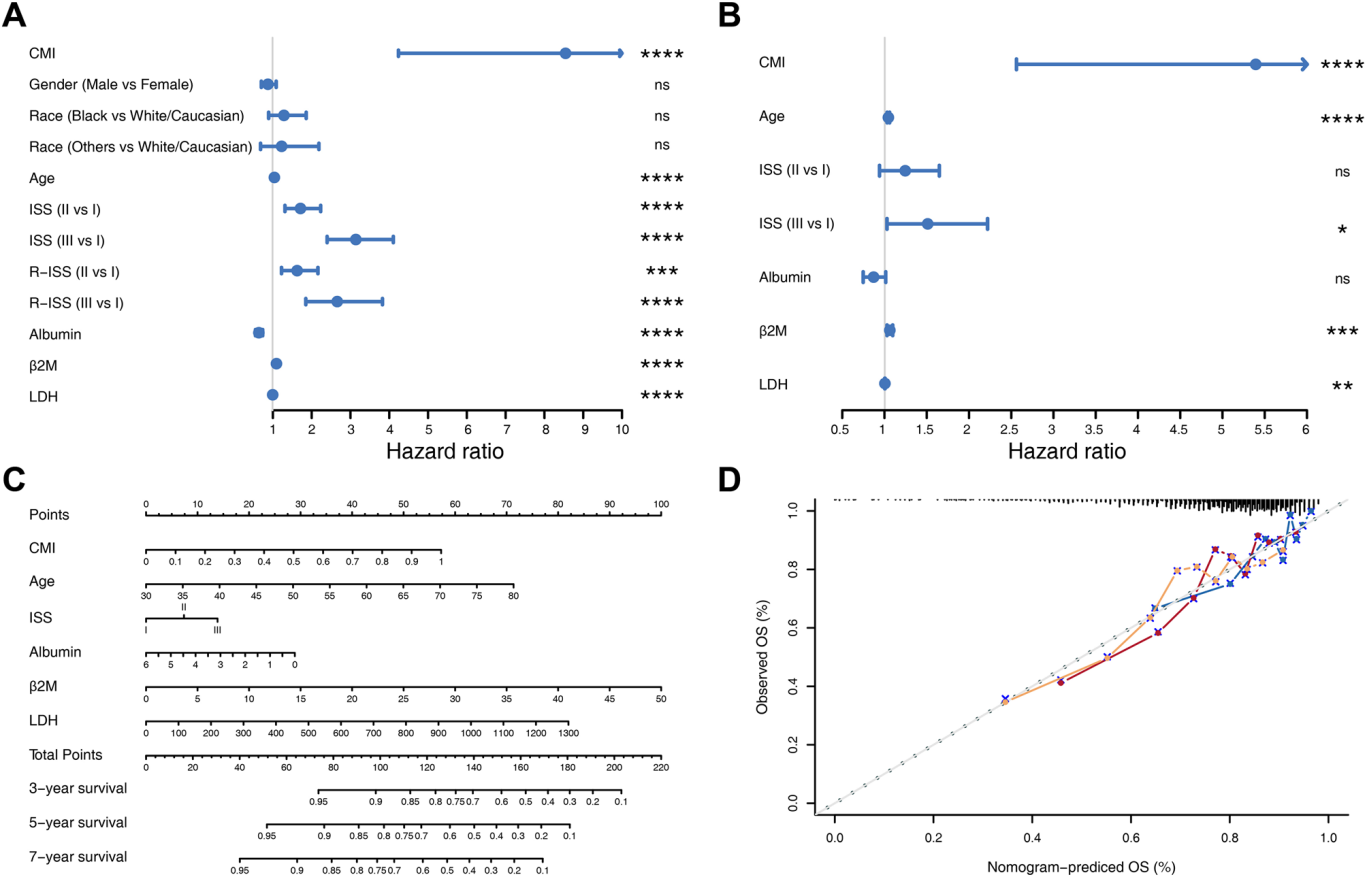
Development and evaluation of cholesterol metabolism-correlated clinicopathologic nomogram. (**A**, **B**) Forest plots showing (**A**) univariate Cox regression analysis and (**B**) multivariate Cox regression analysis of the prognostic signature and clinicopathologic factors. (**C**) Nomogram for predicting 3-, 5-, and 7-year overall survival in MM patients. (**D**) Calibration curve to evaluate the consistency of predicted and actual overall survival.

To determine whether CMI was an independent prognostic indicator of MM, we conducted univariate Cox regression analysis on the GSE136324 dataset. Factors with *P* < 0.05 were included in multivariate Cox regression analysis (Fig. 7A,B). Our findings revealed that age, ISS, albumin, β2M, and CMI were independent risk factors for OS among MM patients. We then developed a clinicopathologic nomogram by combining these variables to predict the survival probability of MM patients (Fig. 7C). By summing up all points and locating them on the bottom scales, we could easily calculate estimated 3-, 5- and 7-year OS probabilities. The calibration plots for the nomogram showed acceptable agreement between predicted estimates and observed outcomes (Fig. 7D). The Harrell's C-index of the nomogram for predicting OS was significantly higher than that of the ISS staging system for OS (0.678 [95% confidence interval (CI) 0.648–0.708] vs. 0.634; [95% CI 0.605–0.664]; *P* < 0.001), indicating that combining CMI with clinical characteristics provided a better prognostic indicator than using only ISS staging system. We further validated the predictive value of nomogram in the external GSE136337 and GSE24080 datasets using the calibration plots (Supplementary Fig. 11). The C-index was also acceptable for 0.677 (95% CI 0.638–0.717) and 0.682 (95% CI 0.637–0.727) in the GSE136337 and GSE24080 respectively.

## Discussion

Reprogramming of metabolism, which includes dysregulated cholesterol metabolism, has been associated with tumor progression and response to treatment in various types of cancer^36^. Cholesterol homeostasis is crucial for the growth and survival of mammalian cells. However, malignant cells require more cholesterol than normal cells for their rapid growth. Abnormal expression of genes involved in cholesterol biosynthesis and uptake has been observed in breast cancer, ovarian cancer, renal cancer, and other types of cancers^24,37,38^. For instance, HMGCR, a rate-limiting enzyme in cholesterol biosynthesis, was upregulated in many tumors; knockdown of HMGCR could impede tumor proliferation and metastasis^39,40^. Several studies have suggested that targeting cholesterol metabolism could be an effective anti-tumor strategy, with targeted inhibition of HMGCR being preliminarily applied to treat cancer patients^36,41,42^. Nevertheless, the specific role played by cholesterol metabolism in MM remains unclear. Therefore, this study aims to develop a novel prognostic signature comprising multiple genes related to cholesterol metabolism for MM patients.

In this study, we screened 6 CMGs (ANXA2, CHKA, NSDHL, PMVK, SCAP and SQLE) based on a systematical analysis using GEO datasets and established and validated a model which could predict the prognosis of MM. There have been many researches focusing on the genes identified in our study, which could verify the reliability of model to some extent. For instance, annexin A2 (ANXA2) is a calcium-regulated membrane-binding protein which could inhibit PCSK9-enhanced LDLR degradation, reduce PCSK9 protein levels via a translational mechanism and compete with LDLR for binding with PCSK9^43,44^. ANXA2 is found to promote MM cell growth, reduce apoptosis in MM cell lines, increase osteoclast formation and have a significant impact on survival of myeloma patients^45^.Choline kinase alpha (CHKA) is the initial enzyme involved in the biosynthesis of phosphatidylcholine and correlated with cholesterol homeostasis^46^. Previous studies have demonstrated that expression levels of CHKA could affect proliferation, metastasis, and survival of ovarian cancer and glioma^47,48^. Inhibition of CHKA expression could also overcome resistance to TRAIL-mediated apoptosis in ovarian cancer cells^47^. NAD(P) dependent steroid dehydrogenase-like (NSDHL) is an enzyme involved in cholesterol biosynthesis and is found to promote breast cancer growth and metastasis, as well as serve as a biomarker for early detection of gastric cancer^49–51^. Phosphomevalonate kinase (PMVK) catalyzes the conversion of mevalonate 5-phosphate to mevalonate diphosphate, a key step in the mevalonate pathway of isoprenoid biosynthesis. Previous researches reveal that PMVK could stabilize β-catenin signaling via mevalonate diphosphate and also associate with responses to chemotherapeutic agents^52,53^. Sterol regulatory element binding protein (SREBP) cleavage-activating protein (SCAP) could bind to SREBPs and mediate their transport from the endoplasmic reticulum to the Golgi in the presence of cholesterol, leading to the regulation of sterol biosynthesis^54^. EGFR signaling in cancer cells could promote N-glycosylation of SCAP by increasing glucose uptake and enhance tumor progression^55^. Squalene epoxidase (SQLE), which is the rate-limiting enzyme catalyzes the stereospecific oxidation of squalene, is one of the most significantly upregulated CMGs in numerous tumors^56^. SQLE promotes the growth and metastasis of various tumors and it’s a potential metabolic target for cancer therapy^57,58^. In together, our study further elucidates the important role of these 6 CMGs in MM, and the prognostic signature constructed by these genes has good performance in predicting the overall survival of MM patients.

Functional analysis revealed that aminoacyle-tRNA biosynthesis, steroid biosynthesis, terpenoid backbone biosynthesis, cholesterol homeostasis, DNA repair, MYC targets and other related pathways were associated with cholesterol metabolism in MM. As an essential component of lipid rafts in mammalian cells, cholesterol is involved in many oncogenic signaling pathways in tumor cells, including MYC, MAPK and Wnt pathway^23^. MYC could enhance cholesterol biosynthesis and dysregulate cholesterol transport and storage, leading to tumor cell progression^59,60^. Previous studies have also reported that there was interaction between cholesterol biosynthesis and DNA repair genes^61,62^. Inhibition of the cholesterol biosynthesis could lead to the accumulation of toxic metabolic intermediates, which causes replicative stress and replication checkpoint-activated cell-cycle arrest. Taken together, our results have reconfirmed the relationship between cholesterol metabolism and signaling pathways.

Bortezomib is now widely used in the treatment of MM, yielding excellent responses. Bortezomib could inhibit the proliferation and induce the apoptosis of MM cells by blocking cytokine circuits, cell adhesion, and angiogenesis^63^. However, not all patients treated with bortezomib experience favorable outcomes. Previous researches have reported that bortezomib resistance might be associated with cholesterol metabolism^64,65^. Our study also revealed that low-CMI patients showed resistant to bortezomib and several other chemotherapeutic agents, demonstrating that cholesterol metabolism in MM might contribute to the response of treatment. Moreover, several researches have suggested that activation of autophagy is associated with bortezomib resistance, while there was minor connection between CMI and autophagy in our research^63,66^.

In addition, immunotherapy has become a promising treatment of cancer in recent years, but how to identify suitable patients for immunotherapy remains unclear^67^. Immune checkpoint inhibitor has been employed in preclinical or clinical trials as a major strategy of immunotherapy. In this study, we chose 6 immune checkpoints and compared the expression levels between the low- and high-CMI groups of MM. The results revealed that they were significantly upregulated in high-CMI group, indicating that MM patients with higher CMI might obtain a better response to immune checkpoint inhibitors. Furthermore, the effect of immunotherapy is closely related to infiltration of immune cells, while cholesterol is found to be associated with number and function of immune cells within tumor microenvironment. Cholesterol could decrease the number of CD8^+^ T cells and cause exhaustion of T cells^68^. Besides, Cancer cells could promote cholesterol efflux from the plasma membrane and induce pro-tumor phenotype of macrophages^24,69^. Consistent with these findings, our study revealed that the high-CMI group has lower CD8^+^ T cells and higher number of M1 macrophages than the low-CMI group. Collectively, the result implicated that MM patients in the high-CMI group may benefit from immunotherapy.

Several limitations still remain in our study. First, we performed the analyses based on public datasets. The performance of the prognostic signature should be further validated by prospective clinical trials. Second, the value and mechanism of 6 signature-contained CMGs in MM remains unclear and needs further in vitro and in vivo experiments to assess. Third, the relationship between the prognostic signature and application of immunotherapy needs to be further explored.

## Conclusions

In conclusion, we constructed and validated a gene signature based on 6 CMGs to predict the prognosis of MM. This gene signature could provide a novel option for the prognosis prediction of MM and help the diagnosis and precision therapy of MM.

### Supplementary Information

Supplementary Information.

## Data Availability

The raw data of our study were downloaded from the GEO database (https://www.ncbi.nlm.nih.gov/geo/).

## Abbreviations

β2M: Beta-2 microglobulin
CIBERSORT: Cell-type identification by estimating relative subsets of RNA transcripts
CI: Confidence interval
C-index: Concordance index
CMG: Cholesterol metabolism-related gene
CMI: Cholesterol metabolism index
DEGs: Differentially expressed gene
FDR: False discovery rate
GEO: Gene expression omnibus
GSEA: Gene set enrichment analysis
ISS: International staging system
KEGG: Kyoto encyclopedia of genes and genomes
LASSO: Least absolute shrinkage and selection operator
LDH: Lactate dehydrogenase
MM: Multiple myeloma
OS: Overall survival
R-ISS: Revised international staging system
