# Supplementary material for: Identification of a cholesterol metabolism-related prognostic signature for multiple myeloma
Source: Sci Rep. 2023 Nov 8;13:19395. doi: 10.1038/s41598-023-46426-z (PMC10632470; doi:10.1038/s41598-023-46426-z)
Supplement: Supplementary file 1 — Supplementary Information. [file 41598_2023_46426_MOESM1_ESM.pdf]

Supplementary Material

Supplementary Table 1. The information of cholesterol metabolism-related genes.

Supplementary Table 2. Univariate Cox regression analysis of cholesterol metabolism-related genes in GSE136324 and GSE136337.

Supplementary Table 3. The combined score of the protein–protein interaction network constructed by STRING database.

Supplementary Figure 1. Venn diagram showing the overlapping genes among CMGs and five multiple myeloma datasets.

Supplementary Figure 2. The correlation matrix plot displaying the correlation features among CMGs included in the signature in the GSE24080 and GSE4204 datasets.

Supplementary Figure 3. Kaplan-Meier curves showing overall survival of MM patients in the high- and low-CMI groups in **(A)** GSE136337, **(B)** GSE24080, **(C)** GSE4204 datasets.

Supplementary Figure 4. Kaplan-Meier curves showing progression-free survival of MM patients in the high- and low-CMI groups in **(A)** GSE136337 and **(B)** GSE24080, and **(C)** event-free survival in GSE4204 datasets.

Supplementary Figure 5. Integrated analysis of prognostic signature and clinicopathologic factors in the GSE136324 dataset. Comparison of normalized CMI according to **(A)** age, **(B)** gender, and **(C)** race. ns, not significant.

Supplementary Figure 6. Integrated analysis of prognostic signature and clinicopathologic factors in the GSE24080 dataset. Comparison of normalized CMI according to **(A)** age, **(B)** gender, **(C)** race, **(D)** albumin levels, **(E)** beta-2 microglobulin (β2M) levels and **(F)** lactate dehydrogenase (LDH) levels. **, $P < 0.01$; ****, $P < 0.0001$; ns, not significant.

Supplementary Figure 7. Integrated analysis of prognostic signature and GEP-70 high-risk status and cytogenetic features in the **(A)** GSE136324 and **(B)** GSE24080 datasets. *, $P < 0.05$; **, $P < 0.01$; ***, $P < 0.001$; ****, $P < 0.0001$.

Supplementary Figure 8. Evaluations of the drug susceptibility between high- and low-CMI groups in the **(A)** GSE136337, **(B)** GSE24080 and **(C)** GSE4204 datasets. *, $P < 0.05$; **, $P < 0.01$; ***, $P < 0.001$; ****, $P < 0.0001$.

Supplementary Figure 9. Summary of GSEA results according to KEGG pathway and hallmark pathway in the GSE24080 dataset.

Supplementary Figure 10. Boxplot showing the comparison of 22 different immune cell types estimated by CIBERSORT between high- and low-CMI groups in (A) GSE136337 and (B) GSE4204 datasets. *, $P < 0.05$; **, $P < 0.01$.

Supplementary Figure 11. Calibration curve to evaluate the consistency of predicted and actual overall survival using **(A)** GSE136337 and **(B)** GSE24080 datasets.

Supplementary Table 1. The list of cholesterol metabolism-related genes.

ABCA1	ARV1	DHCR7	FGF1	LDLRAP1	NR1H4	SOAT1
ABCA2	ATF3	EBP	FMO5	LGALS3	NSDHL	SOAT2
ABCG1	ATF5	ECH1	GGPS1	LGMN	PCSK9	SOD1
ACACB	ATXN2	ELOVL2	GLDC	LPCAT3	PCYT2	SP1
ACADL	AVPR1A	ELOVL3	GNAI1	LPL	PDK3	SQLE
ACADVL	CD9	ELOVL4	GNB3	MAL2	PLAUR	SREBF1
ACAT2	CH25H	ELOVL5	GPAM	MBTPS1	PLSCR1	SREBF2
ACSL1	CHKA	ELOVL6	GPX8	MBTPS2	PMVK	STARD4
ACSL3	CLU	EPHX2	GUSB	MSMO1	PNRC1	STX5
ACSS2	CPEB2	ERLIN1	HMGCR	MVD	PPARG	TM7SF2
ADH4	CTNNB1	ERLIN2	HMGCS1	MVK	PRKAA1	TMEM97
ALCAM	CXCL16	ERRFI1	HMGCS2	MYLIP	RAN	TNFRSF12A
ANTXR2	CYP27A1	ETHE1	IDI1	NFIL3	S100A11	TP53INP1
ANXA13	CYP46A1	FADS2	IDI2	NFYA	SC5D	TRIB3
ANXA2	CYP7A1	FASN	JAG1	NFYB	SCAP	TTC39B
ANXA5	DGAT2	FBXO6	KPNB1	NFYC	SCD	
APOB	DGKQ	FDFT1	LBR	NR1H2	SEMA3B	
APOE	DHCR24	FDPS	LDLR	NR1H3	SERPINA12	

**Supplementary Table 2. Univariate Cox regression analysis of cholesterol metabolism-related genes in GSE136324 and GSE136337.**								
Gene symbol	GSE136324				GSE136337			
	HR	HR.confint.lower	HR.confint.upper	P value	HR	HR.confint.lower	HR.confint.upper	P value
ABCA1	0.955	0.6525	1.398	0.81	0.992	0.7723	1.274	0.95
ABCA2	0.7547	0.5029	1.133	0.17	0.831	0.5483	1.259	0.38
ABCG1	1.06	0.831	1.353	0.64	1.069	0.8071	1.417	0.64
ACACB	0.9725	0.7835	1.207	0.8	0.7413	0.5872	0.9358	0.012
ACADL	0.8508	0.5673	1.276	0.43	0.9461	0.4519	1.98	0.88
ACADVL	1.183	0.9608	1.456	0.11	0.96	0.7749	1.189	0.71
ACAT2	0.7929	0.6137	1.025	0.076	1.056	0.8108	1.375	0.69
ACSL1	0.9532	0.8462	1.074	0.43	0.9734	0.8382	1.13	0.72
ACSL3	1.057	0.8234	1.357	0.66	0.9286	0.7628	1.13	0.46
ACSS2	1.386	1.039	1.849	0.027	1.031	0.7493	1.42	0.85
ADH4	0.5801	0.2709	1.242	0.16	0.8137	0.1933	3.426	0.78
ALCAM	0.9319	0.7366	1.179	0.56	0.8724	0.7648	0.9951	0.042
ANTXR2	0.8793	0.6379	1.212	0.43	0.9044	0.6755	1.211	0.5
ANXA13	0.8641	0.6195	1.205	0.39	0.8612	0.5872	1.263	0.44
ANXA2	1.278	1.054	1.551	0.013	1.339	1.099	1.632	0.0038
ANXA5	1.241	1.034	1.489	0.021	1.004	0.804	1.254	0.97
APOB	0.876	0.6016	1.276	0.49	1.255	0.4834	3.26	0.64
APOE	1.017	0.8709	1.187	0.83	0.8335	0.6933	1.002	0.053
ARV1	1.067	0.8269	1.377	0.62	1.085	0.6633	1.776	0.74
ATF3	1.143	0.8246	1.584	0.42	1.051	0.8725	1.267	0.6
ATF5	1.226	1.062	1.416	0.0055	1.045	0.8591	1.272	0.66

ATXN2	1.281	0.8981	1.826	0.17	0.9703	0.7469	1.26	0.82
AVPR1A	0.8519	0.458	1.585	0.61	1.042	0.6675	1.628	0.85
CD9	1.024	0.889	1.179	0.75	1.076	0.8293	1.395	0.58
CH25H	1.136	0.9241	1.397	0.23	1.068	0.6103	1.87	0.82
CHKA	1.371	1.012	1.858	0.042	1.905	1.231	2.95	0.0038
CLU	0.9809	0.8551	1.125	0.78	1.474	1.078	2.014	0.015
CPEB2	0.8915	0.6191	1.284	0.54	0.9525	0.7062	1.285	0.75
CTNNB1	1.249	0.9716	1.605	0.083	0.7117	0.5026	1.008	0.055
CXCL16	0.8534	0.7023	1.037	0.11	0.707	0.5	0.9997	0.05
CYP27A1	0.8264	0.609	1.122	0.22	1.142	0.7186	1.815	0.57
CYP46A1	0.9732	0.7171	1.321	0.86	0.9221	0.6153	1.382	0.69
CYP7A1	0.6889	0.3348	1.418	0.31	1.107	0.2855	4.293	0.88
DGAT2	1.046	0.8738	1.252	0.63	1.121	0.9057	1.388	0.29
DGKQ	0.9752	0.6969	1.365	0.88	0.8302	0.6154	1.12	0.22
DHCR24	1.2	0.9757	1.475	0.084	1.156	0.9246	1.445	0.2
DHCR7	1.423	1.068	1.897	0.016	1.332	1.025	1.73	0.032
EBP	1.057	0.749	1.491	0.75	1.219	0.8592	1.729	0.27
ECH1	1.12	0.8625	1.453	0.4	1.175	0.8605	1.605	0.31
ELOVL2	0.5999	0.3826	0.9405	0.026	1.17	0.6381	2.146	0.61
ELOVL3	0.7307	0.5677	0.9406	0.015	0.9909	0.5836	1.682	0.97
ELOVL4	1.2	1.036	1.39	0.015	1.01	0.9064	1.126	0.86
ELOVL5	0.7261	0.5668	0.9302	0.011	1.041	0.5522	1.963	0.9
ELOVL6	0.7523	0.5745	0.9851	0.039	1.314	0.95	1.816	0.099
EPHX2	1.31	1.116	1.539	0.00096	0.9454	0.8141	1.098	0.46
ERLIN1	0.8656	0.7061	1.061	0.17	1.206	0.8243	1.764	0.33

ERLIN2	1.088	0.8363	1.416	0.53	0.9046	0.7198	1.137	0.39
ERRFI1	1.017	0.8749	1.181	0.83	0.6302	0.4552	0.8724	0.0054
ETHE1	0.7307	0.5688	0.9386	0.014	0.9656	0.7199	1.295	0.82
FADS2	0.4944	0.3592	0.6805	1.60E-05	0.823	0.4961	1.365	0.45
FASN	0.991	0.7509	1.308	0.95	1.189	0.8115	1.741	0.37
FBXO6	1.268	1.071	1.5	0.0057	1.128	0.953	1.335	0.16
FDFT1	0.7188	0.5753	0.8982	0.0037	1.073	0.8915	1.291	0.46
FDPS	1.304	1.022	1.664	0.033	1.495	1.155	1.935	0.0022
FGF1	1.03	0.6615	1.604	0.9	1.887	1.037	3.433	0.038
FMO5	0.6204	0.3517	1.094	0.099	0.5569	0.2565	1.209	0.14
GGPS1	1.603	1.168	2.202	0.0035	1.264	0.9438	1.692	0.12
GLDC	1.124	0.9816	1.287	0.091	1.044	0.9371	1.163	0.44
GNAI1	1.063	0.9034	1.251	0.46	1.158	0.9411	1.425	0.17
GNB3	0.7408	0.5405	1.016	0.062	0.9206	0.6325	1.34	0.67
GPAM	0.9725	0.8657	1.092	0.64	1.016	0.7758	1.33	0.91
GPX8	1.04	0.8873	1.219	0.63	0.8808	0.5546	1.399	0.59
GUSB	1.506	1.143	1.983	0.0036	1.509	1.033	2.205	0.033
HMGCR	1.256	1.008	1.566	0.043	1.085	0.8722	1.351	0.46
HMGCS1	1.998	1.444	2.766	3.00E-05	1.154	0.8092	1.647	0.43
HMGCS2	0.9466	0.7265	1.233	0.68	0.933	0.6082	1.431	0.75
IDI1	0.8339	0.6375	1.091	0.18	0.9437	0.6265	1.422	0.78
IDI2	0.7879	0.6261	0.9915	0.042	1.2	0.8732	1.65	0.26
JAG1	1.374	1.102	1.713	0.0048	0.7059	0.501	0.9947	0.047
KPNB1	1.264	0.8791	1.818	0.21	1.063	0.8174	1.382	0.65
LBR	0.843	0.7199	0.9872	0.034	1.277	1.071	1.523	0.0065

LDLR	1.24	0.9615	1.599	0.097	1.111	0.884	1.396	0.37
LDLRAP1	1.377	1.096	1.731	0.0061	1.009	0.7854	1.296	0.95
LGALS3	0.8279	0.6907	0.9923	0.041	1.209	0.9217	1.585	0.17
LGMN	0.9926	0.8568	1.15	0.92	1.03	0.926	1.145	0.59
LPCAT3	0.7482	0.5824	0.9613	0.023	0.785	0.4054	1.52	0.47
LPL	1.009	0.896	1.136	0.89	0.8571	0.5396	1.362	0.51
MAL2	1.135	0.9987	1.29	0.052	1.064	0.949	1.193	0.29
MBTPS1	1.394	1.066	1.823	0.015	0.8671	0.6362	1.182	0.37
MBTPS2	1.861	1.227	2.821	0.0034	1.198	0.8928	1.606	0.23
MSMO1	1.302	1.119	1.514	0.00065	0.9959	0.8562	1.158	0.96
MVD	0.8416	0.6142	1.153	0.28	0.8726	0.6181	1.232	0.44
MVK	0.6556	0.4204	1.022	0.063	0.8986	0.5182	1.558	0.7
MYLIP	0.885	0.6832	1.146	0.35	0.9002	0.7403	1.095	0.29
NFIL3	1.248	1.068	1.457	0.0052	0.9866	0.8537	1.14	0.85
NFYA	1.156	0.7737	1.726	0.48	1.091	0.6759	1.762	0.72
NFYB	1.02	0.7801	1.334	0.88	1.111	0.9167	1.348	0.28
NFYC	1.125	0.6969	1.816	0.63	1.027	0.7375	1.431	0.87
NR1H2	1.387	1.037	1.855	0.028	0.9164	0.7201	1.166	0.48
NR1H3	1.177	0.9558	1.449	0.12	0.771	0.564	1.054	0.1
NR1H4	0.451	0.2811	0.7235	0.00096	1.271	0.6047	2.672	0.53
NSDHL	2.124	1.589	2.84	3.70E-07	2.062	1.576	2.698	1.30E-07
PCSK9	0.6425	0.4474	0.9226	0.017	0.9916	0.6389	1.539	0.97
PCYT2	0.9841	0.7077	1.368	0.92	1.079	0.736	1.582	0.7
PDK3	0.8873	0.6172	1.276	0.52	0.633	0.4645	0.8626	0.0038
PLAUR	0.706	0.5569	0.895	0.004	0.8345	0.6116	1.139	0.25

PLSCR1	1.008	0.8646	1.175	0.92	1.028	0.9194	1.149	0.63
PMVK	1.457	1.197	1.773	0.00017	1.473	1.175	1.846	0.00078
PNRC1	1.102	0.9282	1.307	0.27	1.007	0.7918	1.281	0.95
PPARG	0.998	0.8057	1.236	0.99	0.8444	0.5079	1.404	0.51
PRKAA1	0.6497	0.4464	0.9456	0.024	1.042	0.7541	1.44	0.8
RAN	1.294	1.046	1.603	0.018	1.471	1.162	1.862	0.0013
S100A11	0.9775	0.8275	1.155	0.79	1.147	0.9957	1.32	0.057
SC5D	1.079	0.8989	1.296	0.41	0.8167	0.6303	1.058	0.13
SCAP	1.649	1.118	2.433	0.012	1.472	1.059	2.045	0.021
SCD	1.014	0.8365	1.228	0.89	1.112	0.9227	1.341	0.26
SEMA3B	0.8303	0.625	1.103	0.2	1.107	0.7764	1.578	0.57
SERPINA12	0.8573	0.607	1.211	0.38	0.9065	0.507	1.621	0.74
SOAT1	1.416	1.111	1.803	0.0049	1.065	0.9191	1.234	0.4
SOAT2	0.7522	0.558	1.014	0.062	1.007	0.6092	1.665	0.98
SOD1	0.9406	0.7154	1.237	0.66	1.419	1.126	1.789	0.003
SP1	1.012	0.75	1.366	0.94	1.049	0.7308	1.505	0.8
SQLE	1.68	1.291	2.188	0.00012	1.302	1.034	1.639	0.025
SREBF1	0.7387	0.5302	1.029	0.074	0.8366	0.5493	1.274	0.41
SREBF2	1.118	0.8227	1.519	0.48	0.9909	0.7552	1.3	0.95
STARD4	1.274	1.051	1.544	0.014	0.7355	0.5837	0.9267	0.0092
STX5	1.439	1.109	1.867	0.0062	0.9888	0.7511	1.302	0.94
TM7SF2	1.363	1.072	1.733	0.012	0.938	0.7297	1.206	0.62
TMEM97	1.314	1.01	1.71	0.042	1.458	1.231	1.728	1.30E-05
TNFRSF12A	0.9098	0.7086	1.168	0.46	0.9162	0.6779	1.238	0.57
TP53INP1	1.325	1.109	1.582	0.0019	1.103	0.8712	1.395	0.42

TRIB3	1.246	0.8821	1.761	0.21	1.063	0.8462	1.335	0.6
TTC39B	0.8911	0.5867	1.353	0.59	0.7062	0.4105	1.215	0.21

Supplementary Table 3. The combined score of the protein–protein interaction network constructed by STRING database.

#node1	node2	node1_string_id	node2_string_id	combined_score
CHKA	SQLE	9606.ENSP00000265689	9606.ENSP00000265896	0.204
NSDHL	SCAP	9606.ENSP00000359297	9606.ENSP00000265565	0.187
NSDHL	SQLE	9606.ENSP00000359297	9606.ENSP00000265896	0.995
NSDHL	PMVK	9606.ENSP00000359297	9606.ENSP00000357452	0.62
PMVK	SCAP	9606.ENSP00000357452	9606.ENSP00000265565	0.282
PMVK	SQLE	9606.ENSP00000357452	9606.ENSP00000265896	0.715
SCAP	SQLE	9606.ENSP00000265565	9606.ENSP00000265896	0.48

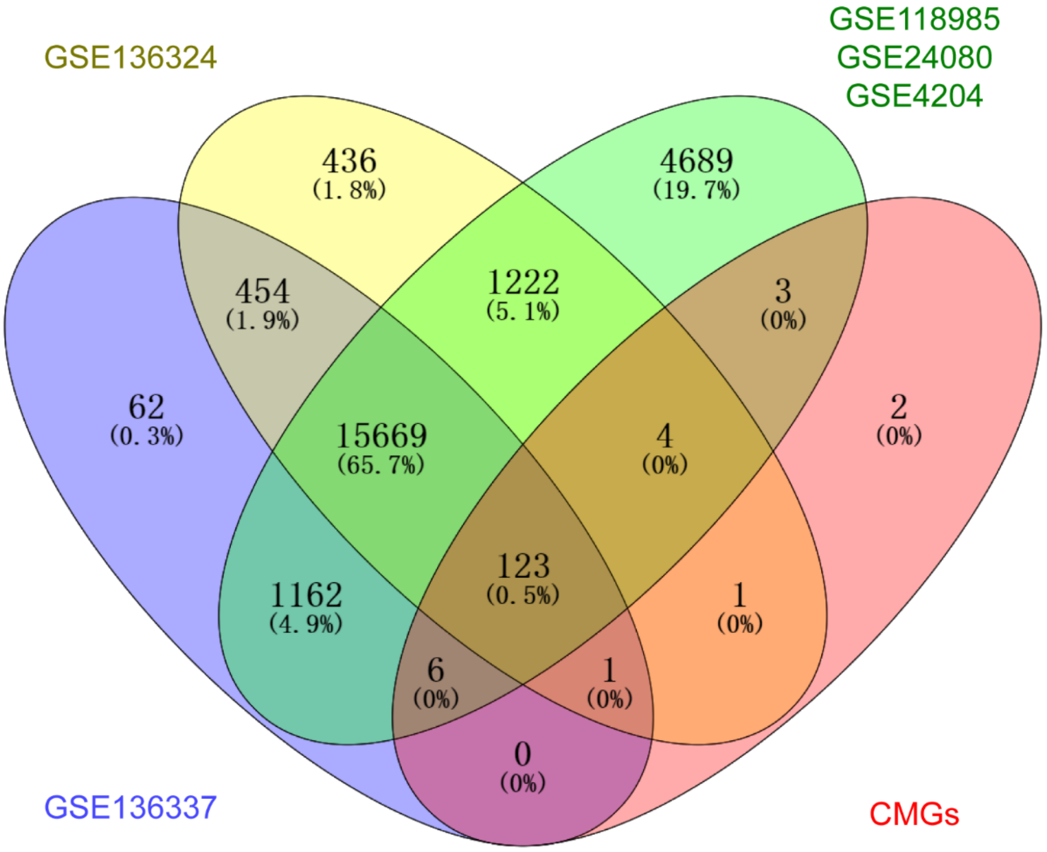

Supplementary Figure 1. Venn diagram showing the overlapping genes among CMGs and five multiple myeloma datasets.

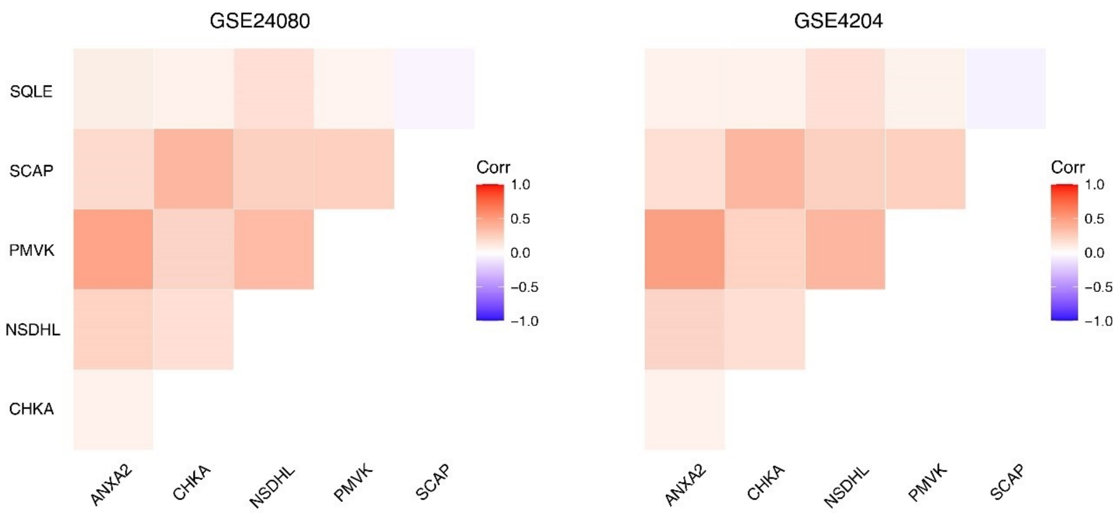

Supplementary Figure 2. The correlation matrix plot displaying the correlation features among CMGs included in the signature in the GSE24080 and GSE4204 datasets.

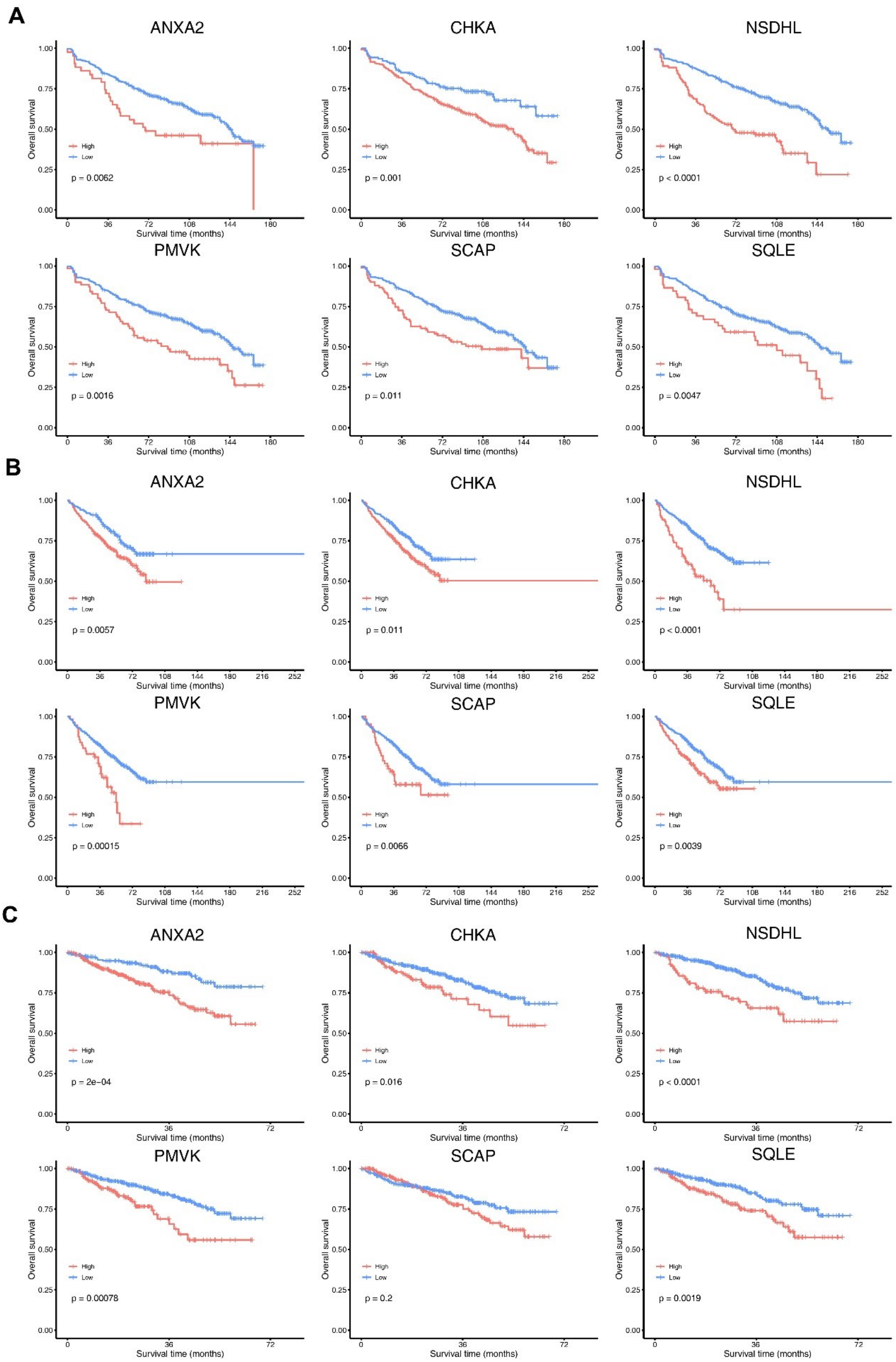

Supplementary Figure 3. Kaplan-Meier curves showing overall survival of MM patients in the high- and low-CMI groups in **(A)** GSE136337, **(B)** GSE24080, **(C)** GSE4204 datasets.

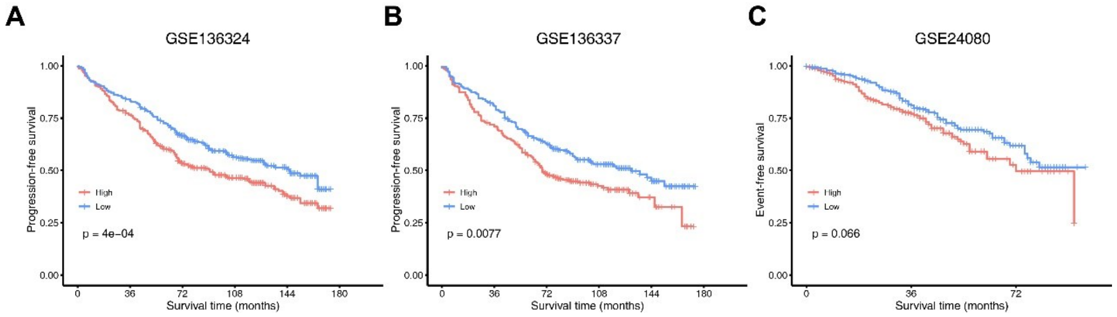

Supplementary Figure 4. Kaplan-Meier curves showing progression-free survival of MM patients in the high- and low-CMI groups in **(A)** GSE136337 and **(B)** GSE24080, and **(C)** event-free survival in GSE4204 datasets.

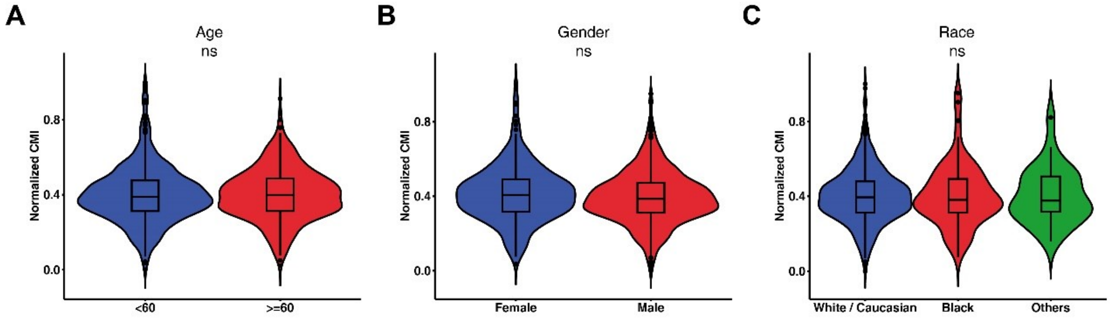

Supplementary Figure 5. Integrated analysis of prognostic signature and clinicopathologic factors in the GSE136324 dataset. Comparison of normalized CMI according to **(A)** age, **(B)** gender, and **(C)** race. ns, not significant.

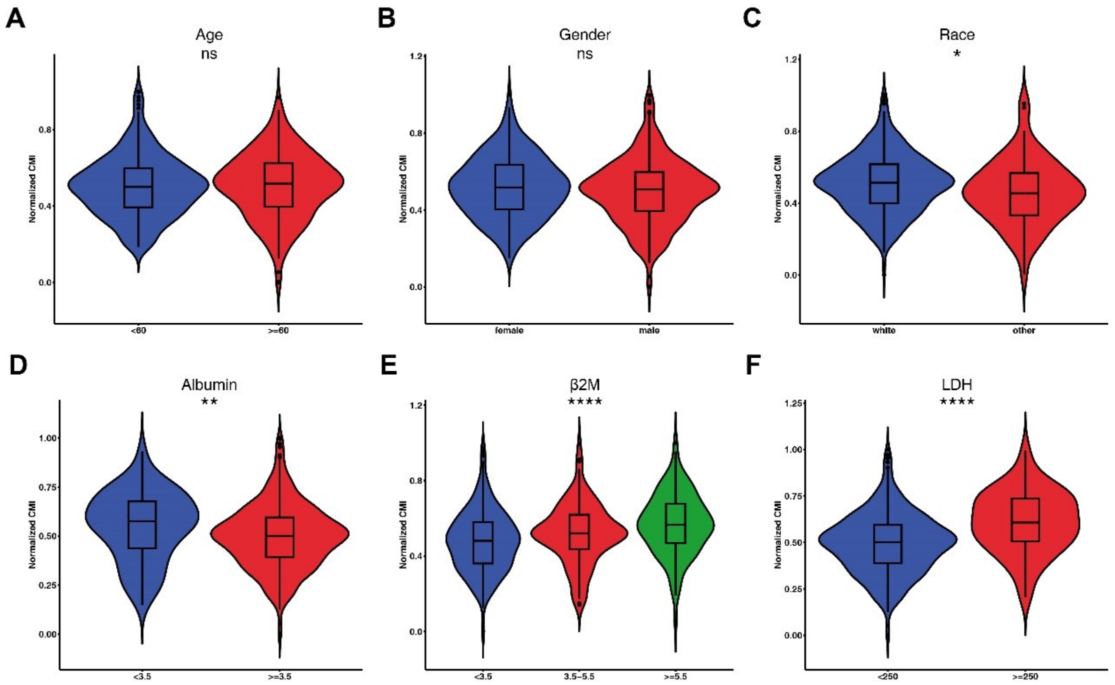

Supplementary Figure 6. Integrated analysis of prognostic signature and clinicopathologic factors in the GSE24080 dataset. Comparison of normalized CMI according to **(A)** age, **(B)** gender, **(C)** race, **(D)** albumin levels, **(E)** beta-2 microglobulin (β2M) levels and **(F)** lactate dehydrogenase (LDH) levels. **, $P < 0.01$; ****, $P < 0.0001$; ns, not significant.

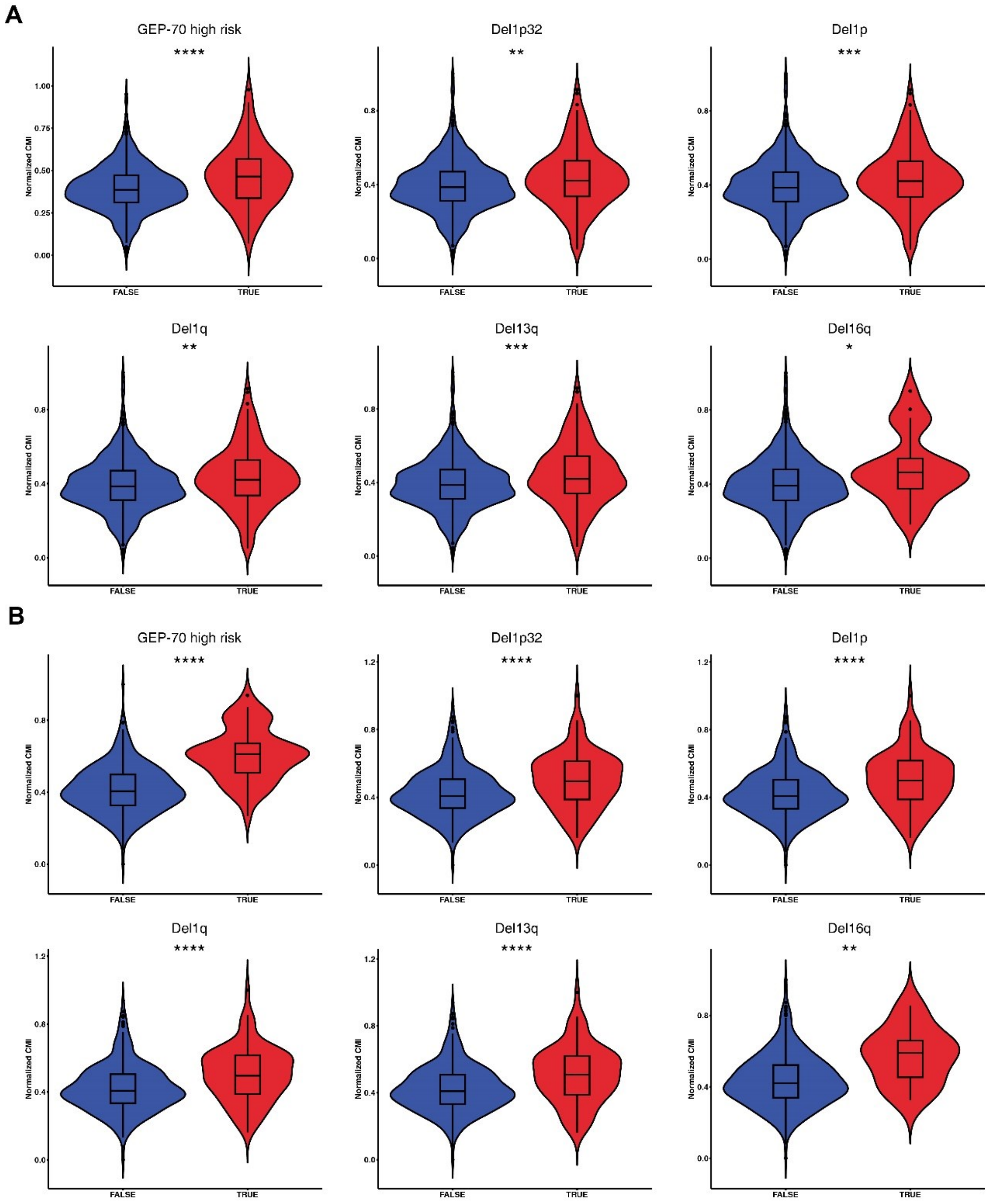

Supplementary Figure 7. Integrated analysis of prognostic signature and GEP-70 high-risk status and cytogenetic features in the **(A)** GSE136324 and **(B)** GSE24080 datasets. *, $P < 0.05$; **, $P < 0.01$; ***, $P < 0.001$; ****, $P < 0.0001$.

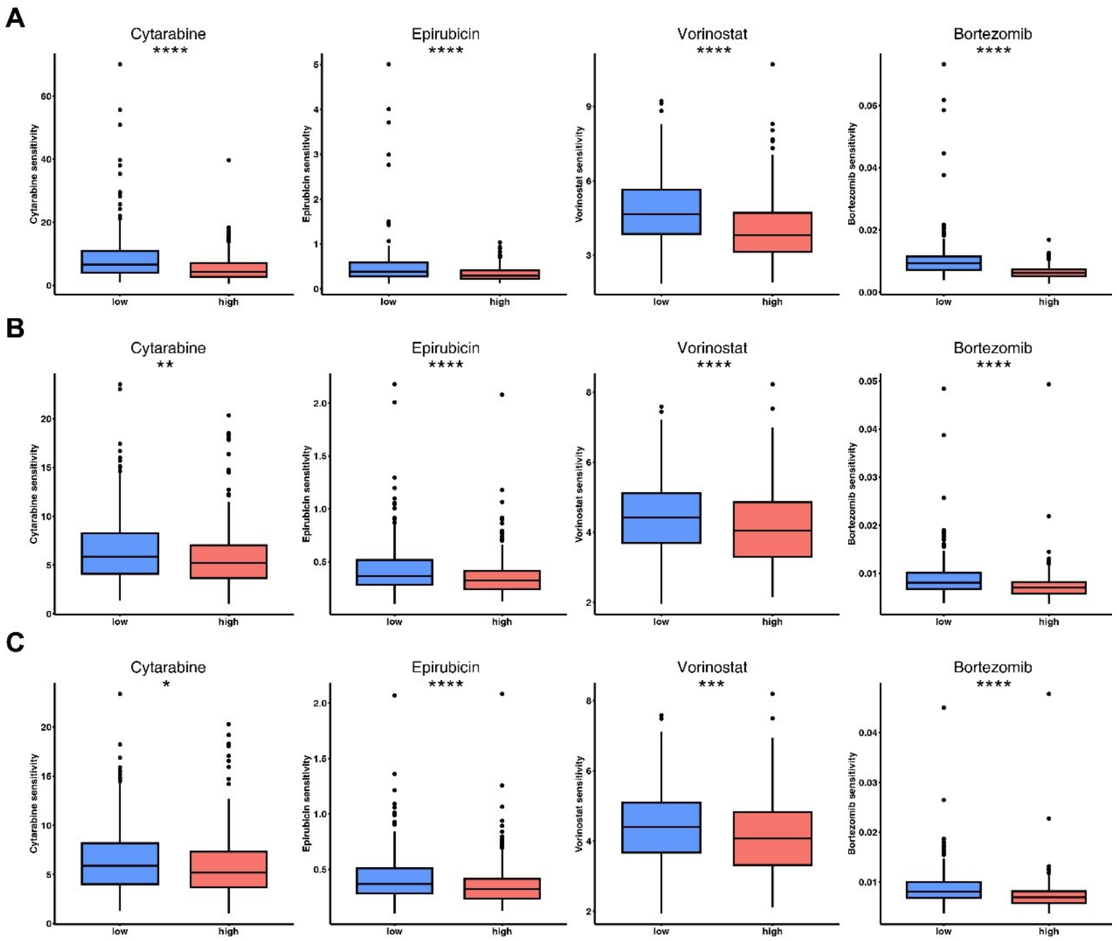

Supplementary Figure 8. Evaluations of the drug susceptibility between high- and low-CMI groups in the **(A)** GSE136337, **(B)** GSE24080 and **(C)** GSE4204 datasets. *, $P < 0.05$; **, $P < 0.01$; ***, $P < 0.001$; ****, $P < 0.0001$.

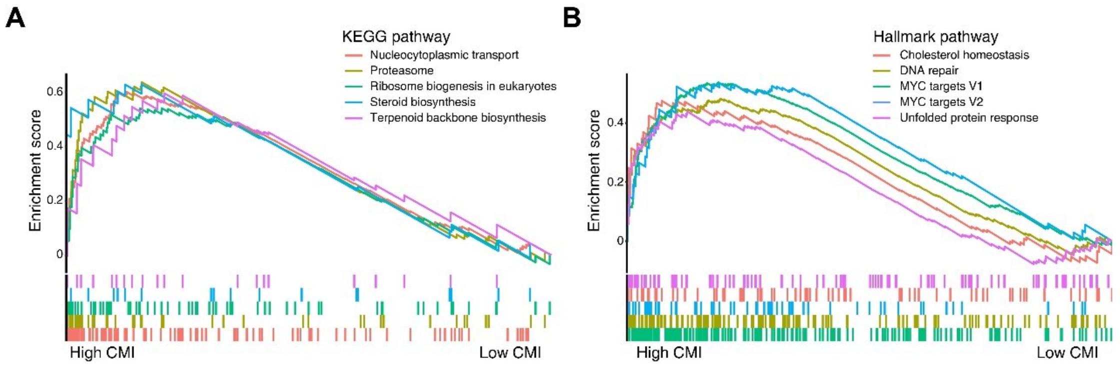

Supplementary Figure 9. Summary of GSEA results according to KEGG pathway and hallmark pathway in the GSE24080 dataset.

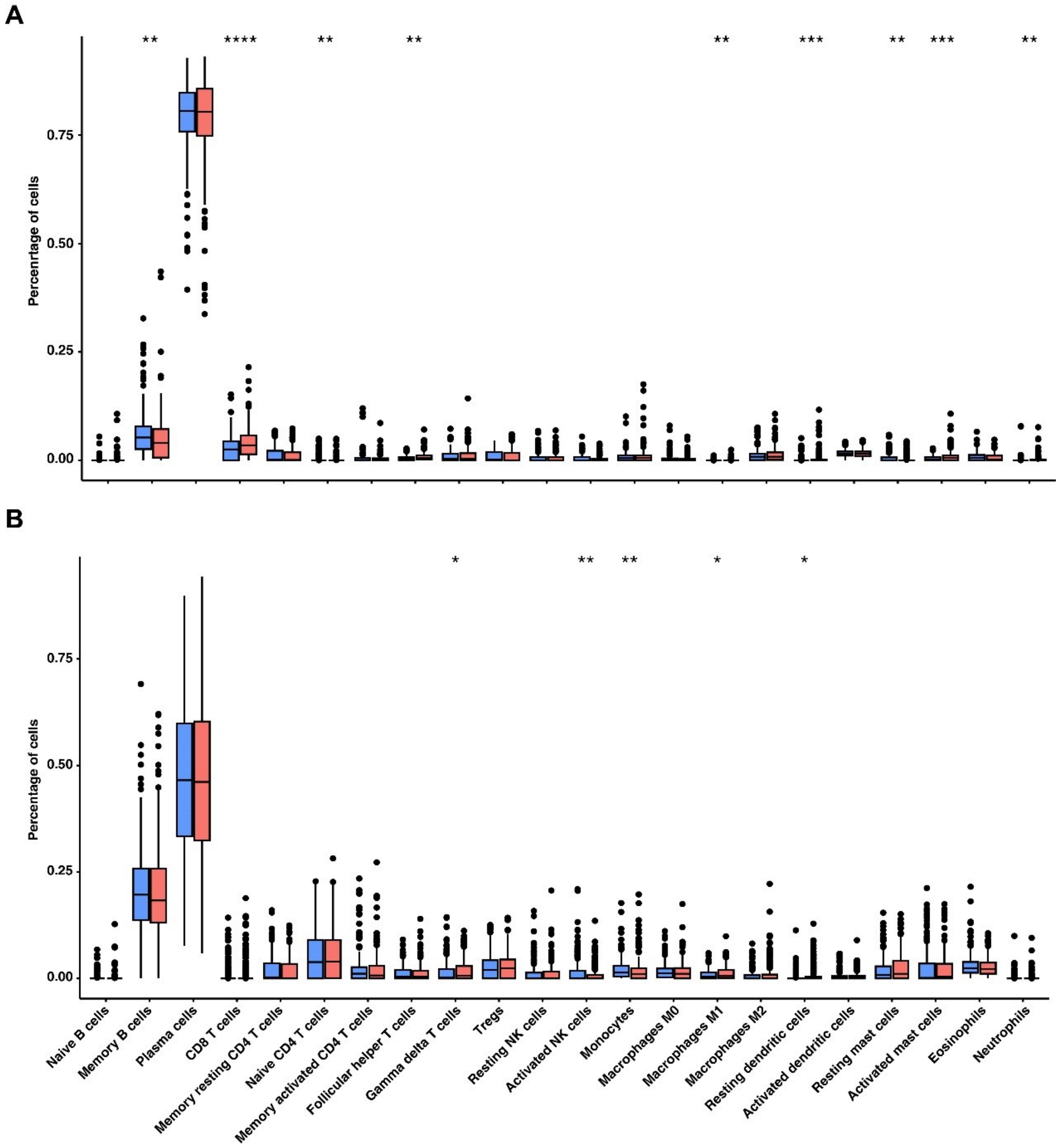

Supplementary Figure 10. Boxplot showing the comparison of 22 different immune cell types estimated by CIBERSORT between high- and low-CMI groups in (A) GSE24080 and (B) GSE4204 datasets. *, P < 0.05; **, P < 0.01.

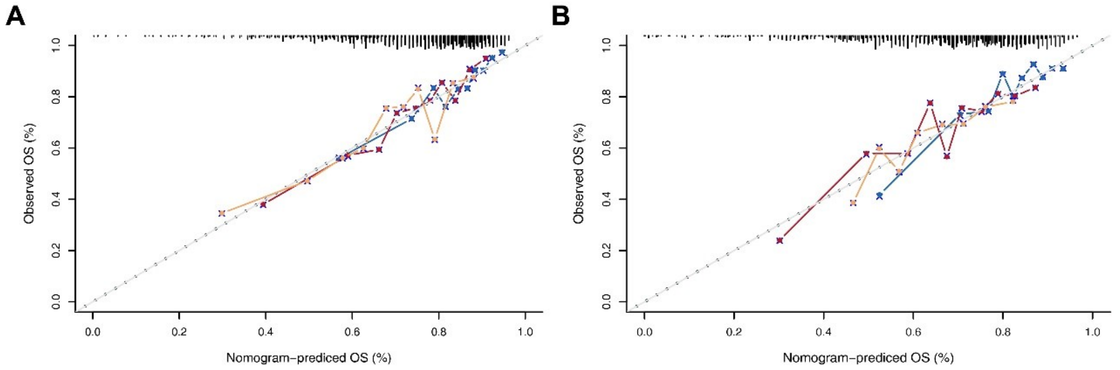

Supplementary Figure 11. Calibration curve to evaluate the consistency of predicted and actual overall survival using **(A)** GSE136337 and **(B)** GSE24080 datasets.
